# Trichuriasis in Differential Diagnosis: A Case of Colonoscopic Identification

**DOI:** 10.14309/crj.0000000000001806

**Published:** 2025-08-11

**Authors:** Ping He, Ankoor H. Patel, Anish Vinit Patel, Ellen Stein

**Affiliations:** 1 Division of Gastroenterology and Hepatology, Rutgers Robert Wood Johnson Medical School, New Brunswick, NJ, USA

**Keywords:** Trichuris trichiura, helminth infection, anemia, eosinophilia, colonoscopy

## CASE REPORT

*Trichuris trichiura*, the whipworm, is a soil-transmitted helminth that can infect the gastrointestinal tract and cause symptoms including vague gastrointestinal complaints, anemia, and eosinophilia. Globally, approximately 465 million people are infected in 2010.^1^ Heavy infections (≥800 worms) pose a substantial risk of anemia, with estimated blood loss of 0.005 mL per worm per day.^2^ We report a patient with iron deficiency anemia, irregular bowel habits, and eosinophilia, where *T. trichiura* infection was diagnosed by colonoscopy.

A 40-year-old woman with a history of hyperlipidemia presented with abdominal pain for 1 year. She described an intermittent, sharp pain in the left upper quadrant radiating to her back lasting 2 days and believed her symptoms associated with her menstrual cycles. She reported taking ibuprofen daily for 3 months. She reported irregular bowel habits alternating between constipation and diarrhea. She took oral iron for longstanding anemia, previously attributed to menorrhagia. She emigrated from Honduras 3 years ago. She denied any pertinent family history or prior endoscopic evaluations. Bloodwork revealed an iron deficiency anemia (Hemoglobin 11.4 g/dL; Iron 22 μg/dL, Iron Saturation 5%, TIBC 424 μg/dL) with peripheral eosinophilia (11.7%; 0.9 × 10^3^/µL). From prior emergency department visit, an ultrasound revealed cholelithiasis and a CT showed distended food-filled stomach and colonic stool burden.

**Figure 1.**
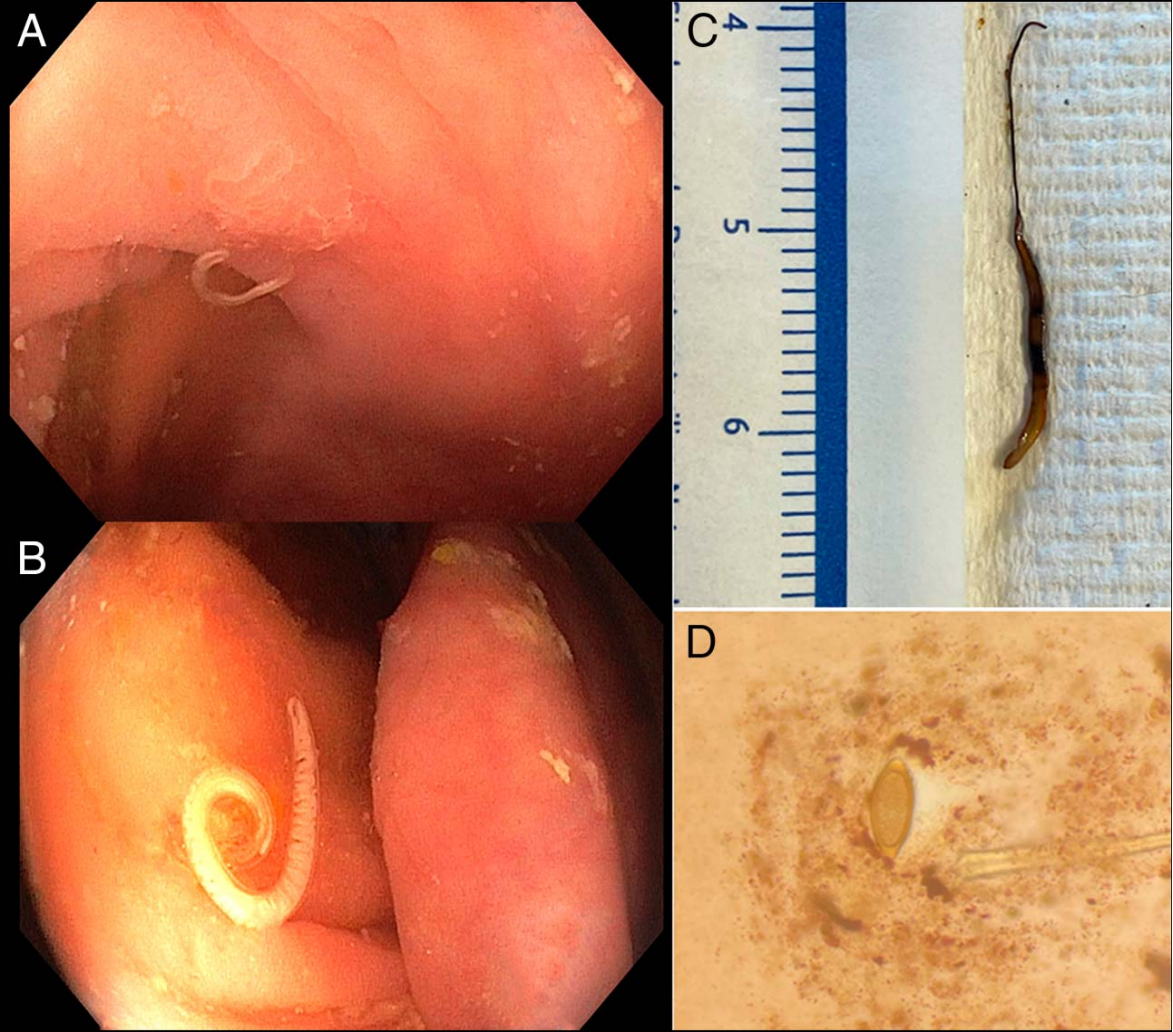
(A) Endoscopic image of *Trichuris trichiura* in its typical location in the cecum, seen next to the appendiceal orifice to the bottom left. (B) Near-focus image of female whipworm next to the ileocecal valve. (C) Pathology sample of the whipworm from (B), extracted endoscopically, with the characteristic “whip-like” anterior end. (D) Whipworm ova seen on stool O&P showing barrel-shaped, thick-shelled characteristics and possess a pair of polar “plugs” at each end.

Upper endoscopy revealed atrophic gastritis and duodenitis with biopsies showing increased intraepithelial lymphocytes without flat villi. Colonoscopy showed live intestinal worms at the cecum (Video, Figure 1). Fluid aspiration in the cecum for ova and parasites returned positive for *Trichuris trichiura* (Figure 1). She completed a 3-day course of albendazole, and eradication was confirmed by repeat testing. Evaluation for autoimmune atrophic gastritis and celiac disease was performed. This case underscores the importance of considering parasitic infections in the differential and recognizing associated endoscopic findings, especially in patients originating from endemic regions.

## DISCLOSURES

Author contributions: All authors worked in all 4 aspects of authorship as per ICMJE guidelines, including drafting, reviewing, investigating, and conceptualization.

Financial disclosure: None to report.

Informed consent was obtained for this case report.
